# Case Report: A *de novo NSD2* multiple exon deletion variant in a child with Rauch-Steindl syndrome

**DOI:** 10.3389/fped.2026.1776647

**Published:** 2026-05-15

**Authors:** Chanchan Li, Mingyu Xie, Qi Peng, Xiaomei Lu, Baimao Zhong

**Affiliations:** 1 The First School of Clinical Medicine, Guangdong Medical University, Zhanjiang, Guangdong, China; 2 Rare Diseases Diagnosis & Treatment Center, Dongguan Children’s Hospital Affiliated to Guangdong Medical University, Dongguan, Guangdong, China; 3 Dongguan Pediatric Research Institute, Dongguan Children's Hospital Affiliated to Guangdong Medical University, Dongguan, Guangdong, China

**Keywords:** autism spectrum disorder, *de novo* variant, NSD2 gene, Rauch-Steindl syndrome, whole-exome sequencing

## Abstract

Rauch-Steindl syndrome (RAUST) is a very rare genetic syndrome caused by pathogenic variants in the NSD2 gene on chromosome 4p16.3. Its clinical phenotype resembles that of Wolf-Hirschhorn syndrome (WHS) but is generally milder. Using whole-exome sequencing (WES), we identified a novel *de novo* deletion spanning exons 6–22 of the NSD2 gene in a 6-year-old Chinese boy diagnosed with RAUST. The patient presented with facial dysmorphisms, language retardation, global developmental delay, autism, and hypotonia. These findings further support the notion that haploinsufficiency of NSD2 is a key mechanism in WHS and that molecular genetic testing provides a more accurate diagnosis for such patients. The novel variant reported here expands the known mutational spectrum of NSD2.

## Background

Rauch–Steindl syndrome (RAUST) (MIM #619695) arises from heterozygous mutations in the *NSD2* gene (MIM #602952) located on chromosome 4p16. Its clinical presentation closely resembles that of Wolf–Hirschhorn syndrome (WHS) (WHS; MIM #194190), albeit generally less severe. WHS is primarily a contiguous gene deletion syndrome involving the heterozygous loss of several genes on 4p16, among which NSD2 is a key contributor. Notably, RAUST typically lacks the classic craniofacial features, such as the “Greek warrior helmet” nasal appearance, and the epileptic manifestations frequently associated with WHS (1, 2). The syndrome is characterized by impaired pre- and postnatal growth, which may include short stature and microcephaly, along with characteristic dysmorphic facial features and variable developmental delay. This delay often encompasses deficits in motor and speech acquisition and can involve mild intellectual impairment. In this study, we identified a novel *de novo* multi-exon loss variant in the *NSD2* gene in a Chinese boy diagnosed with Rauch–Steindl syndrome. Furthermore, our findings contribute to expanding the phenotypic spectrum associated with WHS and the mutational spectrum of *NSD2*.

## Materials and methods

### Study patient

A 6-year-old boy was referred to our neurorehabilitation department for evaluation of delayed language development. Written informed consent was obtained from the proband's parents for the publication of clinical data and genetic results. This study was approved by the institutional review board, and all demographic, clinical, and genetic information was documented using a standardized case report form.

### DNA extraction and whole-exome sequencing

Genomic DNA was extracted from peripheral blood samples obtained from the proband. Whole-exome sequencing (WES) was performed at the Central Laboratory of Genetics and Metabolism. Target enrichment was carried out using the Agilent SureSelect Human All Exon V5 Kit, followed by sequencing on an Illumina HiSeq 2500 platform in accordance with the manufacturer's protocols. For the identification of single nucleotide variants (SNVs) and small insertions or deletions, variant annotation was conducted using the Translational Genomics Expert platform, which integrates the VarElect scoring system for variant prioritization.

For copy number variant (CNV) analysis, we applied DECoN (Detection of Exon Copy Number Variants), a bioinformatic tool specifically designed to detect exon-level CNVs from targeted and whole-exome sequencing data. DECoN identifies potential CNVs by comparing the normalized read depth of each exon in the proband against a reference set of control samples sequenced under identical conditions, employing a beta-binomial model to assess significant deviations. The minimum average read depth per exon was ≥100×, which meets the recommended threshold for reliable CNV detection.

The suspected multi-exon deletion in the *NSD2* gene, as identified by DECoN, was subsequently validated using quantitative real-time PCR (qPCR) with specific primers flanking the deleted exons.

## Results

### Clinical description

The proband, a male infant born to non-consanguineous Han Chinese parents, was delivered via cesarean section at 38 weeks of gestation due to oligohydramnios following an otherwise uncomplicated pregnancy. Reduced fetal movements were noted on ultrasound during the third trimester. His birth weight was 2.3 kg and birth length was 46 cm, both below the 3rd percentile for gestational age and sex, consistent with small for gestational age. Postnatal weight normalized by two months of age. Although he skipped typical sitting and crawling milestones, he achieved independent walking at 18 months and could utter “mom” and “dad” by age 2.

At age 6, the patient underwent a comprehensive developmental reassessment at our hospital. At presentation, his expressive vocabulary remained limited to fewer than 20 words, and he was unable to understand or follow simple commands. Physical examination revealed microcephaly (head circumference: 45 cm; <1st percentile; *Z*-score = –3.0), with height (103 cm; <1st percentile; Z = –3.0) and weight (12.5 kg; <1st percentile; Z = –3.0) significantly below age-appropriate norms based on WHO growth standards. The patient presented with subtle facial dysmorphism, including a broad forehead and an inverted triangular facial shape. The characteristic “Greek warrior helmet” face (defined by a broad nasal root, high arched eyebrows, and upslanted palpebral fissures) was not observed. There was no hypertelorism or epicanthal folds. He exhibited a flat nasal bridge, a long and smooth philtrum, a thin upper lip, and an intact palate. Mild microretrognathia was noted. The ears were normally positioned butshowed mildly simplified morphology. No limb deformities were observed. He exhibited generalized hypotonia affecting all four limbs. Deep tendon reflexes were symmetrically elicited but were brisk. No tremors, ataxia, or choreiform movements were observed. Electroencephalography was normal. Fundoscopic examination revealed normal ocular structures with no evidence of optic nerve atrophy or retinal abnormalities. No cortical visual impairment was detected. Auditory brainstem response testing demonstrated normal bilateral hearing thresholds. Brain magnetic resonance imaging performed with a 3.0T scanner revealedno structural abnormalities.

Neurodevelopmental assessment: Clinical evaluation based on DSM-5 criteria revealed significant impairments in social communication, including inability to imitate, minimal interest in peer interactions, preference for solitary play (though eye contact was preserved), and poor communicative attitude. Restricted and repetitive behaviors were evidenced by food selectivity (preferring soft foods avoiding hard foods) and persistent drooling. The Modified Checklist for Autism in Toddlers, Revised with Follow - Up (M - CHAT - R/F) score was 10 (high risk), and adaptive behavior scale scores were 57, 56, 55, and 58 across domains, indicating moderate to severe adaptive deficits. Based on the integration of clinical observation, DSM-5 criteria, and standardized assessment results, a formal diagnosis of autism spectrum disorder and global developmental delay/intellectual disability was established by an experienced developmental pediatrician.

### Genetic analysis

**Figure 1:**
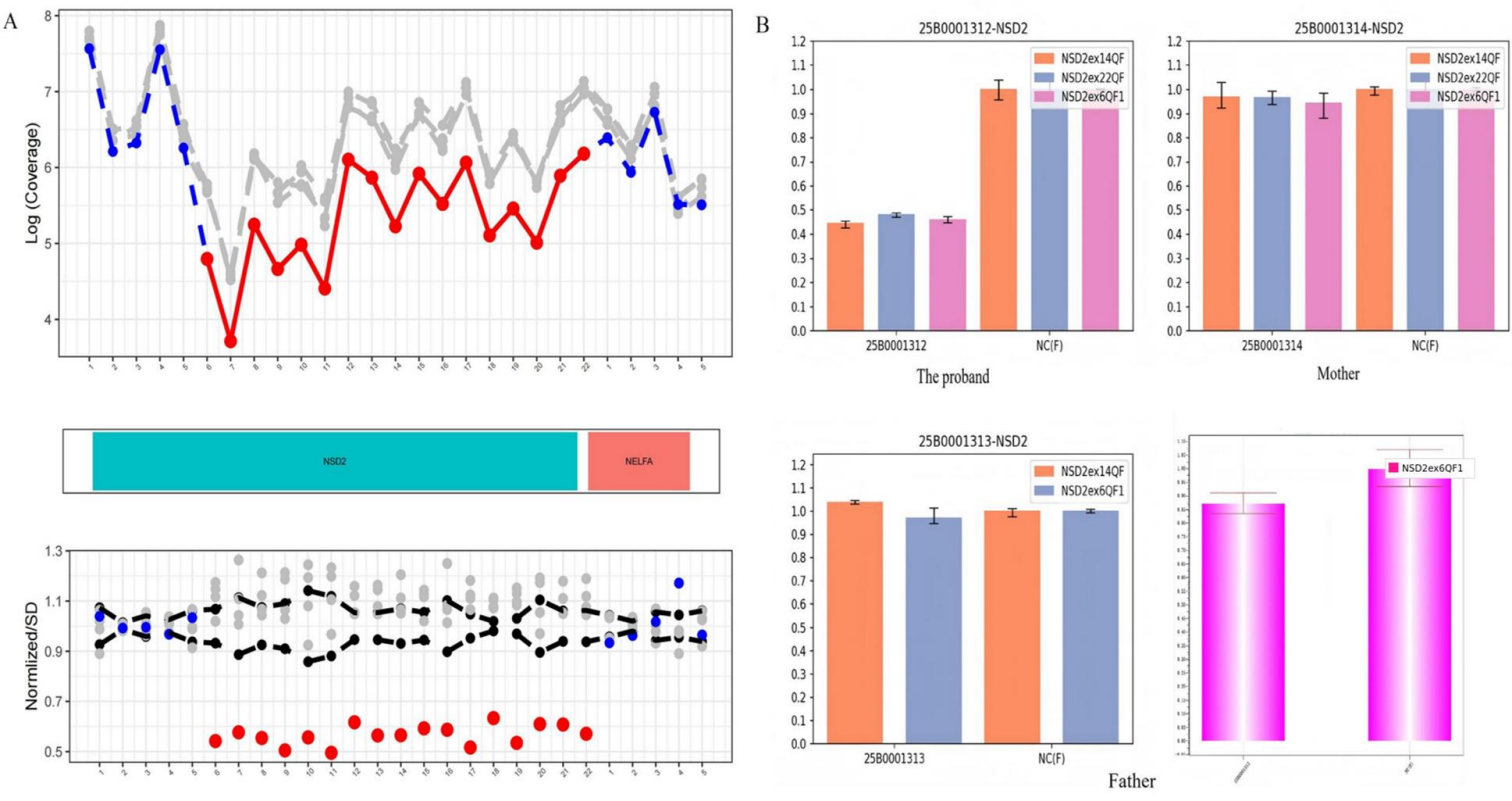
Identification and validation of the *de novo* NSD2 multi-exon deletion. **(A)** Exon-level copy number variant (CNV) analysis using DECoN. The proband (red line/dots) shows a distinct drop in coverage starting from exon 6 through exon 22 of the *NSD2* gene (NM_001042424.3). **(B)** Quantitative real-time PCR (qPCR) validation. The deletion was validated using specific primers for exons 6, 14, and 22. In the proband, the relative copy numbers for all three tested exons are approximately 0.5 compared to the negative control (NC), confirming a heterozygous deletion. In contrast, the results for the mother and father are comparable to the control, indicating the variant is *de novo*.

WES identified a heterozygous *de novo* deletion spanning exons 6–22 of the *NSD2* gene (NM_001042424.3) in the proband.Genomic coordinates for the deletion were mapped to chr4:g.1932354_1980636del (GRCh37). This copy number variant (CNV) was initially detected using a dedicated bioinformatic pipeline (DECoN) and subsequently validated via quantitative real-time PCR (qPCR). The qPCR results confirmed the presence of the multi-exon deletion in the proband and its absence in both biological parents, establishing the *de novo* nature of the variant (Figure 1). The variant has been deposited in ClinVar under accession number SCV007495225.

### Final diagnosis

The patient presented with a constellation of features—including prenatal growth restriction, microcephaly, hypotonia, global developmental delay, and autism spectrum disorder—that prompted a broad differential diagnosis. Initially, Silver-Russell syndrome was considered given the growth restriction and triangular face; however, the absence of body asymmetry made this diagnosis less likely. Wolf-Hirschhorn syndrome was also considered due to the presence of microcephaly and developmental delay, but the lack of seizures, cardiac defects, and the characteristic “Greek warrior helmet” facies argued against a typical 4p16.3 deletion. Microcephalic primordial dwarfism disorders (e.g., Seckel syndrome) were entertained but deemed less probable in the absence of distinctive facial features.

Given the broad differential and non-diagnostic initial workup, whole-exome sequencing (WES) was pursued for its ability to simultaneously detect both single nucleotide variants (SNVs) and copy number variants (CNVs). The identification of a *de novo* heterozygous deletion in *NSD2* established a molecular diagnosis of Rauch-Steindl syndrome (RAUST), thereby resolving the diagnostic uncertainty. This finding explains the core phenotype, including the recently recognized association of *NSD2* haploinsufficiency with ASD. Moreover, the absence of seizures and typical WHS facies further supports a diagnosis of RAUST over Wolf-Hirschhorn syndrome, refining the initial clinical suspicion.

## Discussion and conclusion

The *NSD2* gene (WHSC1), located on chromosome 4p16.3, encodes a nuclear SET domain-containing protein with four key domains: a PWWP domain, an HMG box, a SET domain, and a PHD-type zinc finger. It serves as the principal enzyme responsible for dimethylating histone H3 at lysine 36 (H3K36me2) in most tissues (3, 4), a modification involved in diverse biological processes such as early development, cytokine signaling, DNA damage response, and class switch recombination. Haploinsufficiency of *NSD2*, resulting from gene defects, is established as the primary mechanism underlying RAUST (5). Mutations in *NSD2* are confirmed to cause multi-system abnormalities, including facial dysmorphism, microcephaly, growth retardation, intellectual disability, and language delay (3, 6–10). Murine models corroborate that *NSD2* loss leads to growth restriction, craniofacial malformations, and midline fusion defects (11, 12). Clinically, RAUST phenotypically overlaps with but is milder than WHS; it typically lacks epilepsy, cleft lip/palate, cardiac/renal malformations, and the distinctive “Greek helmet” facial profile (2).

In the previous *NSD2* cohort study, 79% of the *NSD2* variants were *de novo*, and 14% were inherited from an affected parent (2). One third of the patients had missense mutations in the *NSD2* gene, while two-thirds had different truncating variants (2). In our study, large exon deletions are classified as truncation mutations. Similar exon deletions have been previously reported, with varying clinical manifestations (13). One case exhibits mild intellectual disability, while another presents with severe intellectual disability combined with autism (14). Our case study found that the patient with autism had extremely poor life adaptation skills and was unable to complete the intelligence assessment. In the previously reported literature (Supplementary Table S1), almost all cases where the *NSD2* gene had a nonsense mutation or a missense mutation, intellectual disability was present. However, the severity of the intellectual disability was not associated with the type of *NSD2* gene mutation. In the WHS study, all WHS patients presented clinical manifestations of intellectual disability. Specifically, 10% of them had mild intellectual disability, 25% had moderate intellectual disability, and 65% had severe/very severe intellectual disability (15). In the study of the WHS genotype-phenotype correlation, it was found that the severity of intellectual disability is not related to the size of the gene deletion (16).

There have been previous reports on the incidence of autism in patients with RAUST, but the number of cases is relatively limited (17). In our case, the proband was diagnosed with autism at the age of 2. Previous animal experiments have demonstrated that the loss of *NSD2* gene function results in the dysregulation of synaptic genes and alterations in H3K36 dimethylation, which are associated with neurological development disorders (18). This appears to account for the clinical phenotypes of intellectual disability, assessment for language delay, and autism observed in individuals with *NSD2* gene defects. Previously, a report had detailed that they showed delayed bone age, but no signs of growth hormone deficiency. Three cases received growth hormone treatment. Their height showed a slight improvement, and there were no obvious complications (19, 20). Perhaps growth hormone therapy is a means to address short stature.

Nearly all reported patients exhibited manifestations of intrauterine growth restriction (IUGR) and postnatal growth retardation, with the severity varying across individuals. *In vitro* experimental data indicated that mutations in the *NSD2* gene are associated with a decrease in H3K36me2 methylation activity (2). Patients with RAUST also exhibit muscle hypotonia, which may be related to muscle atrophy (20). Almost all RAUST patients exhibit an atypical “Greek warrior helmet” appearance, and the facial deformities are less severe than those of patients with WHS. In addition, the typical characteristics of RAUST patients differ from those of WHS patients. While WHS patients frequently exhibit epileptic symptoms, no cases of such symptoms have been reported in RAUST patients to date. Patients with WHS typically also have malformations of other organs, which result from the cumulative effect of multiple gene deletions (17).

## Conclusions

In conclusion, we identified a *de novo* deletion of exons 6–22 in the *NSD2* gene in a Chinese boy with RAUST via whole-exome sequencing. This loss-of-function variant is established as underlying a syndrome characterized by language development delay and autism. Detailed phenotyping and molecular diagnosis will enhance the understanding of phenotype-genotype correlations for *NSD2* pathogenic variants and related disorders, such as WHS. Furthermore, molecular genetic testing of *NSD2* serves as a valuable tool for the clinical diagnosis and genetic counseling of patients with this condition. The novel pathogenic variant reported here expands the mutational spectrum of the *NSD2* gene.

## Data Availability

The original contributions presented in the study are publicly available. This data can be found here: SCV007495225 (ClinVar).
